# Sleeping and Dietary Factors Associated with Chronic Fatigue Syndrome in Taiwanese Preschoolers

**DOI:** 10.3390/children10071149

**Published:** 2023-06-30

**Authors:** Su-Fen Huang, Hui-Ying Duan

**Affiliations:** 1 Department of Early Childhood Education, National Taitung University, No. 369, Sec. 2, University Road, Taitung City 950309, Taiwan; kou@nttu.edu.tw; 2 Department of Infant and Child Care, National Taipei University of Nursing and Health Sciences, No. 365, Min-Te Road, Taipei 112303, Taiwan

**Keywords:** chronic fatigue syndrome, sleep quality, dietary habit

## Abstract

The purpose of this research was to investigate the sleeping and dietary factors associated with the prevalence of chronic fatigue syndrome among Taiwanese preschoolers. Five-year-old preschoolers were randomly selected using a stratified multistage random cluster sampling method. The parents of the preschoolers completed a questionnaire containing items related to symptoms of fatigue and sleeping and dietary habits among the preschoolers. A total of 1536 valid questionnaires were returned. After obtaining the data, the researchers analyzed them using descriptive statistics and a chi-square test. The following results were obtained: (1) chronic fatigue syndrome was typically indicated by yawning during the day, feeling tired, and appearing sleepy; (2) the preschoolers with high sleep quality, adequate sleeping time, and a regular sleep schedule exhibited a lower degree of fatigue; (3) half of the preschoolers who ate three nutritionally balanced meals a day at a regular time exhibited a lower degree of fatigue. Among the three dimensions studied, fatigue was most strongly associated with the “sleepy and inactive/blunted responses/lacking in energy” dimension, followed by the “difficulty concentrating” dimension, and, finally, the “localized pain” dimension. In this study, the association between sleeping habits and symptoms of fatigue in preschool children was verified. The associations of dietary factors with symptoms of fatigue were not confirmed. It is suggested that parents establish a good sleep schedule for preschool children based on the study findings.

## 1. Introduction

On account of societal changes, children in preschools have been exhibiting eye rubbing and excessive yawning in the morning. These children are sleepy during static discussion sessions and are reluctant to move or play during dynamic activities. Why do preschoolers appear tired so early in the day? One study compared the behaviors of children aged between 3 and 6 years in Taiwan and Japan and observed morning-time sleepiness and fatigue in children in both regions [1]. One study [2] examined the opinions of preschool teachers and parents in Japan, and 90% of the parents believed their children had symptoms of fatigue.

Numerous empirical studies have investigated the causes of Chronic Fatigue Syndrome (CFS) in young children, children, and adolescents [3]. Another study indicated that CFS in children is associated with quality of life and social support [4]. Studies of children and adolescents revealed that inferior sleep quality, an inactive lifestyle, an unbalanced diet, picky eating, sleep deprivation, and a nocturnal lifestyle increase the risk of CFS [5,6,7,8]. However, a systematic review of 25 years of pediatric CFS research [9] revealed the disbelief of clinicians and researchers in the presence of CFS in elementary school students as well as their limited knowledge about symptoms of fatigue experienced by children. Another study analysis of CFS studies conducted in Western countries indicated a CFS prevalence of approximately 0.89% in children and adolescents [10], which varied vastly according to region and age. According to a survey analyzing sleep problems and daytime symptoms of fatigue in preschoolers in a Finnish community [11], 45% of the preschoolers had at least one sleep-related problem occurring at least three times a week.

A study on the association of Japanese preschool children’s habits of late rising and eating brunch on weekends with several physical and mental symptoms [12] revealed that, compared with the children with regular eating and sleeping patterns and schedules, the children with irregular eating and sleeping patterns and delayed schedules had poorer physical and mental conditions. Although the children had regular eating and sleeping patterns and schedules on weekdays, waking up and eating breakfast late on weekends negatively affected the children’s physical and mental conditions. This result indicates the importance of steady and healthy living habits, including regular sleeping, waking up, and eating schedules, for preschool children’s physical and mental health.

According to the recommendations of the American Academy of Sleep Medicine, children aged 3–5 should sleep 10–13 h a day [13]. The research on preschool children in Taiwan, their sleep problems include staying up late, having difficulty falling asleep, and obtaining an insufficient number of hours of nocturnal sleep [14]. Another investigation into the eating behavior of 5-year-old children revealed that, despite the healthy dietary habits of children that have been promoted and related policies that have been implemented in Taiwan over the past 20 years, related practices have yet to be enhanced [15]. According to information on recommended servings of whole grain foods from the 2017–2020 Nutrition and Health Survey in Taiwan [16], the recommended dietary reference intake for boys and girls aged between 1 and 6 years is associated with a slightly lower level of physical activity than that indicated in the Ministry of Health and Welfare’s dietary guidelines for Taiwanese people.

Taiwanese scholars [17] reported the favorable performance of assessment methods for CFS developed internationally after 1990, which quantifiably assess CFS using objective and comprehensive techniques. Scientific researchers analyzed changes in physical functions by observing electromyographic signals, breathing, eyeball movement, and knee reflex [18]. Additionally, an audiocardiographic system was used to measure blood pressure, which facilitates the analysis of bodily fatigue [19]. One study suggested assessing fatigue via blood pressure response to postural change, flicker fusion frequency, change in salivary pH, and the sitting–rising test [20]. On the basis of the literature, each CFS assessment type has its advantages and disadvantages, and the optimal method for determining CFS in children has yet to be identified.

Although determining symptoms of fatigue in children is challenging, the fatigue scale of Chalder et al., developed for adults in 1993 [21], was demonstrated to be applicable to children via the reliability and validity tests of Patel et al. [22]. A study conducted in Japan indicated the high reliability and validity of questionnaires completed by parents reporting their children’s state of fatigue [23]. On the basis of the aforementioned studies, this present study developed a questionnaire by revising a scale developed in Japan. Japanese people and Taiwanese people have many cultural similarities, and the Japanese scale was thus considered an appropriate basis for the tool developed in this current study. The parents of preschool children completed the developed questionnaire, reporting the fatigue profile of their children; these data were used to identify CFS in the children.

The aim of this study is to assess symptoms of fatigue in preschool children and explore the potential presence of CFS in this population. The hypothesis is that preschool children may experience symptoms of fatigue, and the developed composite questionnaire completed by parents can be a reliable tool to assess these symptoms. This study aims to investigate the fatigue profile of preschool children based on parental reports and identify any potential cases of CFS in this population.

In this present study, a survey questionnaire assessing CFS in Taiwanese children and associated factors was developed on the basis of the literature on CFS in children [24]. The researchers then conducted an in-depth investigation of the children’s CFS profiles and analyzed significant differences between factors influencing CFS in children, such as health-related habits (e.g., sleeping and dietary) and daily activities (e.g., outdoor sports, extracurricular activities, and electronic device usage). Effective strategies for improving these factors were proposed. The developed questionnaire can support parents and teachers in the early detection of CFS in children, enabling them to boost children’s physical wellness and energy levels. Moreover, this empirical study raises awareness of the prevalence of CFS in children in Taiwan.

## 2. Methods

Five-year-old preschoolers were randomly selected using a stratified multistage random cluster sampling method. The parents of the preschoolers completed a questionnaire containing items related to symptoms of fatigue and sleeping and dietary habits among the preschoolers. A total of 1536 valid questionnaires were returned. After obtaining the data, the researchers analyzed them using descriptive statistics and a chi-square test.

### 2.1. Subjects and Sampling

#### 2.1.1. Enrollees

This study was reviewed and approved by the Human Research Ethics Committee of National Cheng Kung University, Taiwan, on 22 October 2019. Stratified multistage random cluster sampling was used to select children attending registered public or private preschools in Taiwan during the 2020–2021 school year. Preschoolers with particular physical conditions or weakness, illness, or systemic chronic disease were excluded; a total of 1536 preschoolers aged 5 to 6 years were enrolled and comprised 777 boys (50.59%) and 759 girls (49.41%).

#### 2.1.2. Sample Size

The 194,317 students aged between 5 and 6 years registered as attending public or private preschools in Taiwan according to the Education Statistics for the 2020–2021 school year [25] were adopted as the parent population for estimating the sample size, which was expressed as *n* = Z^2^ × p (1 − p)/ε^2^; with the 95% confidence coefficient as the confidence norm of the sampling design and the sampling error set to 3%, the minimal sample size required was approximately 1061. According to Sudman [26], for general survey research, the recommended average sample size ranges from 1500 to 2500 for studies on a national scale. On the basis of the aforementioned considerations, a sample size of 2000 children were calculated for this study. In public and private preschools in Taiwan, a senior class generally comprises 20 children. In this study, the number of preschools to be sampled (*n* = 100) was obtained by dividing 2000 (sample size of the children) by 20 (number of preschoolers per class). Therefore, approximately 2000 children from 100 preschools were sampled.

#### 2.1.3. Stratified Multistage Random Cluster Sampling

Stratified multistage random cluster sampling was used to divide the 359 townships or cities and districts into 13 strata, including one stratum divided according to ethnicity (“Hakka”) and three strata divided according to geographic location (“mountainous,” “eastern,” and “outlying islands”); for the remaining nine strata, Taiwan was first divided into northern, central, and southern regions, and from each of these regions, three strata were further derived on the basis of population density. Probability proportional to size sampling was used to sample public and private preschools from each stratum, resulting in a total of 104 preschools being sampled. Simple random sampling was then applied to sample 20 children from each of the sampled preschools of students aged between 5 and 6 years, resulting in a total of 2080 children being sampled from around Taiwan.

Participant recruitment involved a random sampling approach, where preschools were selected. Subsequently, the researchers contacted the selected preschools via phone to inquire about their willingness to participate. The researchers personally explained the purpose, target population, and content of this study to the preschools and provided them with a letter of invitation and an explanation of this study. Once the preschools confirmed their participation, a formal request was made to the willing preschools. A sealed envelope containing a parent invitation letter and a questionnaire with clear instructions was handed to the parents of children who were at least five years old in the respective classes. The parents were requested to fill out the questionnaire and return it in a sealed envelope by their child to the preschool teacher. Finally, the questionnaires were collected by the researcher or an assistant. In cases where preschools, teachers, or parents declined to assist with this study or refused to complete the questionnaire, the researchers fully respected their decisions.

#### 2.1.4. Questionnaire Administration and Retrieval

A total of 104 public and private preschools were sampled. A total of 2080 consent forms and 2080 copies of the questionnaire on symptoms of fatigue in children and their associated factors were distributed; 1820 consent forms and 1820 questionnaire copies were returned. Following the removal of invalid questionnaires, a total of 1536 valid questionnaires were retrieved, yielding a valid response rate of 78.7%. The response rate of over 75% has reached the goal of this study, which represents the population to be studied.

### 2.2. Measurements

For this study, a composite questionnaire was developed to assess symptoms of fatigue in preschool children. The advantages of questionnaires are low cost and easy to distribute; thus, they were considered a suitable method. However, because of children’s limited cognitive skills and comprehension of the meanings of items and text, assessing symptoms of fatigue in children using a self-reporting questionnaire was inappropriate. If the parents provide answers based on their observations of their children, it can help overcome the disadvantage of limited cognitive skills and difficulties with self-reporting in preschool children.

The survey questionnaire used in this study comprised a demographic data section, an assessment scale for symptoms of fatigue in children, a survey on children’s health-related habits (sleeping and dietary), and a survey on children’s daily activities (outdoor sports, extracurricular activities, and electronic device usage). Because of length limitations, the daily activities of children will be analyzed in a future study.

#### 2.2.1. Assessment Scale for Symptoms of Fatigue in Children

To analyze the findings of this study in relation to those of studies conducted in other countries, instead of developing a scale assessing symptoms of fatigue in children in Mandarin, this study adopted the ten-item scale assessing symptoms of fatigue in children developed by Japanese scholars Hattori et al. [24] in 2011. The scale has favorable internal consistency, reliability, and applicability for analyzing CFS in children.

For this study, symptoms of fatigue in children were measured on the basis of three dimensions, namely “sleepy and inactive/blunted responses/lacking in energy,” “difficulty concentrating,” and “localized pain.” In particular, the “sleepy and inactive/blunted responses/lacking in energy” dimension comprised four items, namely “My child often yawns during the day,” “My child often looks sleepy,” “My child becomes tired easily,” and “My child seldom engages in physical play.” The “difficulty concentrating” dimension comprised four items, namely “My child often becomes irritated,” “My child often has difficulty sitting still,” “My child often has difficulty concentrating when playing,” and “My child often has difficulty remaining calm.” The “localized pain” dimension comprised two items, namely “My child often experiences headaches and stomach pain” and “My child often looks unwell.” Each of the ten items was rated on a 5-point Likert scale, with scores of 1, 2, 3, 4, and 5 indicating “never,” “rarely,” “sometimes,” “very often,” and “always,” respectively. The total score ranged from 10 to 50; higher scores indicated that the children exhibited a higher degree of fatigue, whereas lower scores indicated that the children exhibited a lower degree of fatigue. The parents provided answers on the basis of their observations of their children. For the questions on symptoms of fatigue in children please see the Appendix A. For the questions on children’s sleep quality please see Appendix B. 

#### 2.2.2. Profile of Children’s Health-Related Habits Questionnaire

This study investigated children’s health-related habits by focusing on two dimensions, namely “preschoolers’ sleep quality” and “preschoolers’ dietary habits”. The composite Children’s Health-Related Habits Questionnaire was developed to measure these dimensions and comprised the Children’s Sleep Profile Questionnaire, designed in reference to Lo and Pan [27], and the Children’s Diet Profile Questionnaire developed in reference to Chen et al. [28]. The two referenced questionnaires were revised, organized, and analyzed in relation to the research foci of this study to formulate items based on a nominal scale. The parents choose answer options [“almost” (positive) or “hardly” (negative)] according to their observations of their children. For the questions on children’s dietary habits please see Appendix C. 

To analyze the validity of the questionnaires developed in this study, pretest questionnaires were formulated, and the items were evaluated by a panel of five experts and scholars to ensure the appropriate wording and relevance of the items. The panel provided suggestions on the addition, removal, and combination of items and on other aspects. After the pretest questionnaires were retrieved, the phrasing of some items and the questionnaire structure were modified according to the panel’s opinions to clarify item meanings, render the phrases fluent, and further improve the structure. Test–retest reliability was adopted to measure the reliability of the questionnaire. Random sampling was used to sample 200 parents of children from 30 public and private preschools in Taitung County, all of whom submitted a test and retest with a 4-week interval. The test–retest reliability of the Assessment Scale for Symptoms of Fatigue in Children and the Children’s Health-Related Habits Questionnaire was 0.92 and 0.94, respectively, and both reached a significance level (*p* < 0.05).

### 2.3. Data Analyses

A statistical test was conducted employing the degree of fatigue in children as the explanatory variable and the profiles of factors, including sleep and dietary habits, as response variables. The dependent variable becomes “hardly” (positive) and “almost”(negative). Descriptive statistics and a chi-square test were conducted; associations with CFS in children were identified, and their statistical difference was analyzed. To determine the overall degree of fatigue in preschoolers, the total scores of the Assessment Scale for Symptoms of Fatigue in Children were used to divide the children into three groups, namely “high score,” “middle score,” and “low score” using the extreme group approach [29]. The total scores were arranged in descending order; the top 27% of scores indicated the high-score group identified as having a high degree of fatigue; the bottom 27% indicated the low-score group identified as having a low degree of fatigue; and the scores between these two groups indicated the middle-score group with a moderate degree of fatigue. When there is a significant difference in the statistical test results, the Bonferroni correction Z Test is performed for post hoc comparison.

## 3. Results

### 3.1. Overview of Assessment of Symptoms of Fatigue in Children

A total of 1536 preschoolers aged 5 to 6 years were enrolled and comprised 777 boys (50.59%) and 759 girls (49.41%). In terms of the level of education of the parents, the majority had a junior college or college degree (*n* = 857; 55.79%). The majority of the parents had an annual household income between TWD 500,000 and TWD 1,140,000 (*n* = 813; 52.93%).

Table 1 presents the profile of SF in preschool children according to the observations of their parents. The highest degree of fatigue observed in children was in the “sleepy and inactive/blunted responses/lacking in energy” dimension, followed by the “difficulty concentrating” dimension, and the lowest degree was observed in the “localized pain” dimension. The items indicating the highest to the lowest degrees of fatigue in children were, in order, “often yawns during the day,” “becomes tired easily,” “often looks sleepy,” “often has difficulty remaining calm,” “seldom engages in physical play,” “often has difficulty sitting still,” “often becomes irritated,” “often has difficulty concentrating when playing,” “often experiences headaches and stomach pain,” and “often looks unwell.” The frequencies and percentages obtained from grouping the total scores of the Assessment Scale for SF in Children using the extreme group approach were 436 (28.38%), 717 (46.67%), and 383 (24.93%).

### 3.2. Analysis of Differences in the Sleep Quality of Children with Varying Degrees of Fatigue

Significant differences in their associations with the degree of fatigue in the children were observed in the following items: “ability to fall asleep within 15 min” [*χ*^2^(2) = 11.82, *p* = 0.003 < 0.05], “sleep through the night” [*χ*^2^(2) = 8.82, *p* = 0.012 < 0.05], “no enuresis during sleep” [*χ*^2^(2) = 8.16, *p* = 0.017 < 0.05], “no sleepwalking” [*χ*^2^(2) = 10.56, *p* = 0.005 < 0.01], “nocturnal sleep for 10–11 h on weekdays [*χ*^2^(2) = 22.88, *p* < 0.0001], “nocturnal sleep for 10–11 h on weekends and holidays” [*χ*^2^(2) = 9.64, *p* = 0.008 < 0.01], “going to bed before 9 p.m. on weekdays” [*χ*^2^(2) = 21.98, *p* < 0.0001], “going to bed before 9 p.m. on weekends and holidays” [*χ*^2^(2) = 15.30, *p* < 0.0001], “waking up before 7 a.m. on weekdays” [*χ*^2^(2) = 11.03, *p* = 0.004 < 0.01], “waking up before 7 a.m. on weekends and holidays” [*χ*^2^(2) = 9.33, *p* = 0.009 < 0.01], and “regular sleep schedule” [*χ*^2^(2) = 17.24, *p* < 0.0001]. The results of the post hoc comparison revealed that, for the children with a low degree of fatigue, the “almost” response was provided for the following items related to sleep quality: “ability to fall asleep within 15 min,” “sleep through the night,” “no sleepwalking,” “nocturnal sleep for 10–11 h on weekdays,” “nocturnal sleep for 10–11 h on weekends and holidays,” “going to bed before 9 p.m. on weekdays,” “going to bed before 9 p.m. on weekends and holidays,” “waking up before 7 a.m. on weekdays,” “waking up before 7 a.m. on weekends and holidays,” and “regular sleep schedule,” whereas no significant differences were observed for “no sleep talking” [*χ*^2^(2) = 6.04, *p* = 0.051 > 0.05] and “no sleep terrors” [χ^2^(2) = 5.73, *p* = 0.057 > 0.05] (Table 2).

### 3.3. Analysis of Differences in the Dietary Habits of Children with Varying Degrees of Fatigue

Significant differences in their associations with the degree of fatigue in the children were observed in the following items: “My child eats three meals of a consistent quantity punctually every day” [*χ*^2^(2) = 15.90, *p* < 0.0001], “My child chews slowly” [*χ*^2^(2) = 10.18, *p* = 0.006 < 0.01], “My child eats at least three servings of vegetables every day” [*χ*^2^(2) = 11.24, *p* = 0.004 < 0.01], “My child drinks at least 1500 mL of boiled water every day” [*χ*^2^(2) = 11.61, *p* = 0.003 < 0.01], “My child avoids Western-style fast foods such as hamburgers and French fries” [*χ*^2^(2) = 12.58, *p* = 0.002 < 0.01], “My child avoids instant noodles and other instant foods” [*χ*^2^(2) = 6.61, *p* = 0.037 < 0.05], “My child avoids processed foods” [*χ*^2^(2) = 15.44, *p* < 0.0001], and “My child avoids fried or creamy foods and other fat-rich foods” [*χ*^2^(2) = 19.05, *p* < 0.0001]. The results of the post hoc comparison revealed that, for the children with a significantly lower degree of fatigue, the “almost” response was provided for the following items relating to dietary habits: “My child eats three meals of a consistent quantity punctually every day,” “My child chews slowly,” “My child eats at least three servings of vegetables every day,” “My child drinks at least 1500 mL of boiled water every day,” “My child avoids Western-style fast foods such as hamburgers and French fries,” “My child avoids instant noodles and other instant foods,” “My child avoids processed foods,” and “My child avoids fried or creamy foods and other fat-rich foods.” No significant differences were observed in the following items: “My child eats breakfast every day, [*χ*^2^(2) = 3.98, *p* = 0.137 > 0.05; OR(95%C.I.): 1.082(0.708–1.655), *p* = 0.714]” “My child avoids eating late-night snacks, [χ^2^(2) = 4.89, *p* = 0.087 > 0.05; OR(95%C.I.): 0.952(0.734–1.235), *p* = 0.710]” “My child is not a picky eater,” [χ^2^(2) =5.82, *p* = 0.054 > 0.05; OR(95%C.I.): 0.828(0.644–1.064), *p* = 0.140] “My child eats foods from the six major groups every day, [χ^2^(2) = 6.22, *p* = 0.051 > 0.05; OR(95%C.I.): 1.078(0.820–1.417), *p* = 0.589]” “My child eats at least two fruits every day,” [χ^2^(2) = 6.29, *p* = 0.051 > 0.05; OR(95%C.I.): 0.998(0.764–1.303), *p* = 0.986] “My child avoids eating snacks,” [χ^2^(2) = 6.00, *p* = 0.051 > 0.05; OR(95%C.I.): 1.005(0.726–1.391), *p* = 0.976] and “My child avoids sodas and sugar-sweetened beverages” [χ^2^(2) = 4.59, *p* = 0.101 > 0.05; OR(95%C.I.): 1.005(0.726–1.391), *p* = 0.976] (Table 3).

## 4. Discussion

According to the results of this study, among the three dimensions relating to CFS in children in Taiwan, “sleepy and inactive/blunted responses/lacking in energy” was more frequently observed in children by their parents, followed by “difficulty concentrating” and “localized pain.”

Regarding the items related to CFS in children, the items with higher scores were “My child often yawns during the day,” “becomes tired easily,” and “often looks sleepy.” Similarly, Yoneyama et al. [3] reported that approximately 40% of Japanese children feel irritated and tired during the day. In the United Kingdom, from pediatric epidemiology, Crawley [30] reported that CFS is relatively common in children and has a wide range of effects on the child, their family, and health care systems, but the majority of children can overcome this illness with specialist treatment. Research into this complicated illness is hindered by small sample sizes, varying definitions, and the lack of a coordinated approach.

By inference, children’s CFS is probably not a single illness; this inference is supported by the results on items (e.g., “My child often experiences headaches and stomach pain” and “often looks unwell” in the “localized pain” dimension) representing less common symptoms of fatigue. Different phenotypes likely underlie different biological pathways and require different treatment approaches.

The results of this study revealed that a low degree of fatigue in the children corresponded to the following items: “being able to fall asleep within 15 min,” “sleep through the night,” “no enuresis during sleep,” “no sleepwalking,” “nocturnal sleep for 10–11 h,” “going to bed before 9 p.m.,” “waking up before 7 a.m.,” and “regular sleep schedule.” These findings are consistent with those of other studies [2,5,6,7,8,22,24,29], which implied a lower degree of fatigue in children who sleep and wake up early every day and who have favorable sleep quality, a sufficient number of hours of sleep, and a regular sleep schedule.

In Simola et al.’s multiple regression analysis, difficulties in initiating and maintaining sleep were most strongly associated with tiredness in the morning and during the day. Poor sleep quality is associated with morning and daytime tiredness [9]. Therefore, regulating the bedtime and waking time of children and ensuring they sleep for a sufficient number of hours are necessary. According to the mainstream preschool daily schedule in Taiwan, students arrive at 8 a.m. However, some of the parents noted that their children were inactive and yawned during the day, indicating that health education related to children’s healthy sleeping and waking behaviors has yet to be implemented in families.

The results of this study revealed that a low degree of fatigue in the children was associated with the following items: “My child eats three meals of a consistent quantity punctually every day,” “chews slowly,” “eats at least three servings of vegetables every day,” “drinks at least 1500 mL of boiled water every day,” “avoids Western-style fast foods such as hamburgers and French fries,” “avoids instant noodles and other instant foods,” “avoids processed foods,” and “eats fried or creamy foods and other fat-rich foods.” These findings are consistent with those of relevant studies.

Hattori et al. [24] reported elevated degrees of fatigue in children who frequently eat instant food, dine out, eat boxed meals, do not have a regular eating schedule, and eat with fewer people. Yoneyama et al. [3] observed elevated degrees of fatigue in children without regular dietary habits, implying that proper and regular dietary habits are effective for eliminating CFS in children.

No significant differences were observed for the following items: “My child eats breakfast every day,” “avoids eating late-night snacks,” “is not a picky eater,” “eats foods from the six major groups every day,” “eats at least two fruits every day” “avoids eating snacks,” and “avoids sodas and sugar-sweetened beverages.”

The absence of significant associations with CFS in children may be attributable to the following factors: parents in Taiwan consider breakfast to be a critical meal, breakfasts are provided in the morning in preschools, and preschool regulations stipulate that foods from the six major groups and fruits must be included in the meals to ensure nutritional balance.

However, because a considerable range of beverages and snacks are available in the 24 h convenience stores abound in Taiwan, young parents are generally habituated to purchasing sugar-sweetened beverages and snacks, which are often irresistible to children. Other parents maintained that their children were picky eaters. Regarding the inconsistent findings relating to the association between such dietary habits and CFS in children, snack-related items, whether the term “snacks” refers to healthy snacks such as nuts, which have high nutritional value, or to sweets such as chocolate, they can cause loss of appetite. Additionally, parents habituated to sugar-sweetened beverages are relatively unlikely to prevent their children from drinking such beverages.

Above all, the following results were obtained: (1) yawning during the day, feeling tired, and appearing sleepy are the three highest chronic fatigue syndromes; (2) preschoolers with high sleep quality, adequate sleeping time, and a regular sleep schedule exhibited a lower degree of fatigue; (3) half of the preschoolers who ate three nutritionally balanced meals a day at a regular time exhibited a lower degree of fatigue.

## 5. Conclusions and Suggestions

According to the results of this study, “sleepy and inactive/blunted responses/lacking in energy” was the CFS-related dimension most frequently exhibited in the children, followed by “difficulty concentrating.” “Localized pain” was exhibited to a lower degree. The correlation between sleeping habits and CFS in children has been confirmed, but no consistent results were obtained in terms of the correlation between dietary habits and CFS in children. It is suggested that parents use their child’s childhood, a critical period for habit formation, to instill healthy sleep and dietary habits in their children to reduce their fatigue and to benefit their health condition. There is no information about children’s sleep duration except a positive or negative response to “Nocturnal sleep for 10–11 h on weekends and holidays” since this study limits that to asking parents to provide some estimate of nightly sleep duration, and the goal of this analysis was to examine causes for fatigue. The Children’s Health-Related Habits Questionnaire only allowed parents to answer “Almost” or “Hardly”. This binary response does not allow the researchers much response variability for subsequent analysis. This is another study limitation. Moreover, on the basis of the results and insights gained throughout the research process, this study proposed recommendations for future research. The potential of this research has confirmed that young children also have fatigue problems, which need to be prevented early. Research into this illness is hampered by small sample sizes, varying definitions, and the lack of a coordinated approach. First, theories could be applied to investigate and identify additional associated factors, such as parental stress and behavior. Second, this study adopted a cross-sectional study design, which precluded the determination of any chronological associations between the variables and the occurrence of CFS, and the causality must be further investigated.

## Figures and Tables

**Table 1:** Results of assessment for symptoms of fatigue in children N = 1536.

Dimension	Item	Never	Rarely	Sometimes	Very Often	Always	Mean	Standard Deviation	Standardized Score	Sequence
		Percentage Represented by Parents’ Response Scores (%)					(M) (SD)		(%)	
Sleepy and inactive/blunted responses/lacking in energy	My child seldom engages in physical play	23.1	16.3	27.4	31.4	1.7	2.72 (1.18)		54.4	5
	My child often yawns during the day	18.8	2.0	37.4	41.9	0.0	3.02 (1.09)		60.4	1
	My child often looks sleepy	18.9	2.6	39.3	39.1	0.0	2.99 (1.08)		59.8	3
	My child becomes tired easily	16.9	4.2	39.6	39.2	0.1	3.01 (1.06)		60.2	2
Difficulty concentrating	My child often becomes irritated	22.9	19.8	28.7	27.6	1.0	2.64 (1.14)		52.8	7
	My child often has difficulty remaining calm	21.7	15.7	30.6	30.4	1.6	2.75 (1.15)		55.0	4
	My child often has difficulty concentrating when playing	24.7	17.9	28.1	28.5	0.8	2.63 (1.16)		52.6	8
	My child often has difficulty sitting still	25.4	16.5	24.4	32.3	1.4	2.68 (1.21)		53.6	6
Localized pain	My child often experiences headaches and stomach pain	30.1	14.8	28.4	26.6	0.1	2.52 (1.18)		50.4	9
	My child often looks unwell	29.3	15.1	32.4	23.2	0.0	2.49 (1.14)		49.8	10
Assessment Scale for Symptoms of Fatigue in Children		23.2	12.5	31.6	32.0	0.7	2.74 (1.14)		54.9	

Standardized score index = (average of the scores of the items/the number of items with full scores) × 100%.

**Table 2:** Homogeneity test of the factors influencing sleep quality in children with varying degrees of fatigue.

Item			Group by Degree of Fatigue Number of Times and Percentage			Chi-Square	*p*-Value	Post Hoc Comparison
	Degree		High-Score Group (A)	Middle-Score Group (B)	Low-Score Group (C)			
Ability to fall asleep within 15 min	Hardly	Count (ratio in %)	216 (14.06)	402 (26.17)	175 (11.39)	11.82 (2)	0.003 **	B > C
		Modified residual	−1.03	3.26	−2.68			
	Almost	Count (ratio in %)	220 (14.32)	315 (20.51)	208 (13.54)			C > B
		Modified residual	1.03	−3.26	2.68			
Sleep through the night	Hardly	Count (ratio in %)	62 (4.04)	111 (7.23)	35 (2.28)	8.82 (2)	0.012 *	B > C
		Modified residual	0.49	2.08	−2.91			
	Almost	Count (ratio in %)	374 (24.35)	606 (39.45)	348 (22.66)			C > B
		Modified residual	−0.49	−2.08	2.91			
No sleep talking	Hardly	Count (ratio in %)	221 (14.39)	377 (24.54)	171 (11.13)	6.04 (2)	0.051	N.S.
		Modified residual	0.31	1.84	−2.45			
	Almost	Count (ratio in %)	215 (14.00)	340 (22.14)	212 (13.80)			
		Modified residual	−0.31	−1.84	2.45			
No enuresis during sleep	Hardly	Count (ratio in %)	174 (11.33)	322 (20.96)	139 (9.05)	8.16 (2)	0.017 *	B > C
		Modified residual	−0.72	2.66	−2.32			
	Almost	Count (ratio in %)	262 (17.06)	395 (25.72)	244 (15.89)			C > B
		Modified residual	0.72	−2.66	2.32			
No sleep terrors	Hardly	Count (ratio in %)	176 (11.46)	297 (19.34)	131 (8.53)	5.73 (2)	0.057	N.S.
		Modified residual	0.53	1.58	−2.37			
	Almost	Count (ratio in %)	260 (16.93)	420 (27.34)	252 (16.41)			
		Modified residual	−0.53	−1.58	2.37			
No sleep walking	Hardly	Count (ratio in %)	152 (9.90)	285 (18.55)	115 (7.49)	10.56 (2)	0.005 **	B > C
		Modified residual	−0.55	2.91	−2.78			
	Almost	Count (ratio in %)	284 (18.49)	432 (28.13)	268 (17.45)			C > B
		Modified residual	0.55	−2.91	2.78			
Nocturnal sleep for 10–11 h on weekdays	Hardly	Count (ratio in %)	267 (17.38)	402 (26.17)	172 (11.20)	22.88 (2)	<0.0001 ***	A > C
		Modified residual	3.22	0.97	−4.47			
	Almost	Count (ratio in %)	169 (11.00)	315 (20.51)	211 (13.74)			C > A
		Modified residual	−3.22	−0.97	4.47			
Nocturnal sleep for 10–11 h on weekends and holidays	Hardly	Count (ratio in %)	205 (13.35)	304 (19.79)	139 (9.05)	9.64 (2)	0.008 **	A > C
		Modified residual	2.41	0.16	−2.7			
	Almost	Count (ratio in %)	231 (15.04)	413 (26.89)	244 (15.89)			C > A
		Modified residual	−2.41	−0.16	2.7			
Going to bed before 9 p.m. on weekdays	Hardly	Count (ratio in %)	340 (22.14)	496 (32.29)	242 (15.76)	21.98 (2)	<0.0001 ***	A > C
		Modified residual	4.21	−0.81	−3.45			
	Almost	Count (ratio in %)	96 (6.25)	221 (14.39)	141 (9.18)			C > A
		Modified residual	−4.21	0.81	3.45			
Going to bed before 9 p.m. on weekends	Hardly	Count (ratio in %)	368 (23.96)	572 (37.24)	281 (18.29)	15.30 (2)	<0.0001 ***	A > C
		Modified residual	3	0.26	−3.43			
	Almost	Count (ratio in %)	68 (4.43)	145 (9.44)	102 (6.64)			C > A
		Modified residual	−3	−0.26	3.43			
Waking up before 7 a.m. on weekdays	Hardly	Count (ratio in %)	267 (17.38)	448 (29.17)	201 13.09)	11.03 (2)	0.004 **	B > C
		Modified residual	0.81	2.13	−3.29			
	Almost	Count (ratio in %)	169 (11.00)	269 (17.51)	182 11.85)			C > B
		Modified residual	−0.81	−2.13	3.29			
Waking up before 7 a.m. on weekends	Hardly	Count (ratio in %)	336 (21.88)	498 (32.42)	264 17.19)	9.33 (2)	0.009 **	A > C
		Modified residual	3.05	−1.68	−1.96			
	Almost	Count (ratio in %)	100 (6.51)	219 (14.26)	119 (7.75)			C > A
		Modified residual	−3.05	1.68	1.96			
Regular sleep schedule	Hardly	Count (ratio in %)	157 (10.22)	220 (14.32)	87 (5.66)	17.24 (2)	<0.0001 ***	A > C
		Modified residual	3.12	0.38	−3.69			
	Almost	Count (ratio in %)	279 (18.16)	497 (32.36)	296 (19.27)			C > A
		Modified residual	−3.12	−0.38	3.69			

Variables of Significance (* *p* ≤ 0.05, ** *p* ≤ 0.01, *** *p* ≤ 0.001); N.S. = not significant (*p* > 0.05).

**Table 3:** Homogeneity test of the factors influencing dietary habits of children with varying degrees of fatigue.

Item			Groups by Degree of Fatigue Number of Times and Percentage			Chi-Square	*p*-Value	Post Hoc Comparison
	Degree		High-Score Group (A)	Middle-Score Group (B)	Low-Score Group (C)			
My child eats breakfast every day	Hardly	Count (ratio in %)	42 (2.73)	64 (4.17)	23 (1.50)	3.98 (2)	0.137	N.S.
		Modified residual	1.1	0.7	−1.95			
	Almost	Count (ratio in %)	394 (25.65)	653 (42.51)	360 (23.44)			
		Modified residual	−1.1	−0.7	1.95			
My child eats three meals of a consistent quantity punctually every day	Hardly	Count (ratio in %)	90 (5.86)	133 (8.66)	41 (2.67)	15.90 (2)	<0.0001 ***	A > C
		Modified residual	2.26	1.32	−3.88			
	Almost	Count (ratio in %)	346 (22.53)	584 (38.02)	342 (22.27)			C > A
		Modified residual	−2.26	−1.32	3.88			
My child chews slowly	Hardly	Count (ratio in %)	312 (20.31)	483 (31.45)	234 (15.23)	10.18 (2)	0.006 **	A > C
		Modified residual	2.4	0.29	−2.83			
	Almost	Count (ratio in %)	124 (8.07)	234 (15.23)	149 (9.70)			C > A
		Modified residual	−2.4	−0.29	2.83			
My child avoids eating late-night snacks	Hardly	Count (ratio in %)	165 (10.74)	282 (18.36)	125 (8.14)	4.89 (2)	0.087	N.S.
		Modified residual	0.31	1.59	−2.15			
	Almost	Count (ratio in %)	271 (17.64)	435 (28.32)	258 (16.80)			
		Modified residual	−0.31	−1.59	2.15			
My child is not a picky eater	Hardly	Count (ratio in %)	281 (18.29)	498 (32.42)	241 (15.69)	5.82 (2)	0.054	N.S.
		Modified residual	−1.02	2.37	−1.67			
	Almost	Count (ratio in %)	155 (10.09)	219 (14.26)	142 (9.24)			
		Modified residual	1.02	−2.37	1.67			
My child eats foods from the six major groups every day	Hardly	Count (ratio in %)	189 (12.30)	310 (20.18)	138 (8.98)	6.222 (2)	0.051	N.S.
		Modified residual	0.94	1.31	−2.49			
	Almost	Count (ratio in %)	247 (16.08)	407 (26.50)	245 (15.95)			
		Modified residual	−0.94	−1.31	2.49			
My child eats at least three servings of vegetables every day	Hardly	Count (ratio in %)	252 (16.41)	442 (28.78)	196 (12.76)	11.24 (2)	0.004 **	B > C
		Modified residual	−0.07	2.75	−3.1			
	Almost	Count (ratio in %)	184 (11.98)	275 (17.90)	187 (12.17)			C > B
		Modified residual	0.07	−2.75	3.1			
My child eats at least two fruits every day	Hardly	Count (ratio in %)	281 (18.29)	476 (30.99)	225 (14.65)	6.29 (2)	0.051	N.S.
		Modified residual	0.27	1.88	−2.44			
	Almost	Count (ratio in %)	155 (10.09)	241 (15.69)	158 (10.29)			
		Modified residual	−0.27	−1.88	2.44			
My child drinks at least 1500 mL of boiled water every day	Hardly	Count (ratio in %)	261 (16.99)	446 (29.04)	198 (12.89)	11.61 (2)	0.003 **	B > C
		Modified residual	0.47	2.45	−3.32			
	Almost	Count (ratio in %)	175 (11.39)	271 (17.64)	185 (12.04)			C > B
		Modified residual	−0.47	−2.45	3.32			
My child avoids eating snacks	Hardly	Count(ratio in %)	320 (20.83)	531 (34.57)	258 (16.80)	6.00 (2)	0.051	N.S.
		Modified residual	0.66	1.52	−2.44			
	Almost	Count (ratio in %)	116 (7.55)	186 (12.11)	125 (8.14)			
		Modified residual	−0.66	−1.52	2.44			
My child avoids sodas and sugar-sweetened beverages	Hardly	Count (ratio in %)	229 (14.91)	393 (25.59)	184 (11.98)	4.59 (2)	0.101	N.S.
		Modified residual	0.02	1.72	−2			
	Almost	Count (ratio in %)	207 (13.48)	324 (21.09)	199 (12.96)			
		Modified residual	−0.02	−1.72	2			
My child avoids Western-style fast foods such as hamburgers and French fries	Hardly	Count (ratio in %)	283 (18.42)	460 (29.95)	208 (13.54)	12.58 (2)	0.002 **	B > C
		Modified residual	1.52	1.99	−3.54			
	Almost	Count (ratio in %)	153 (9.96)	257 (16.73)	175 (11.39)			C > B
		Modified residual	−1.52	−1.99	3.54			
My child avoids instant noodles and other instant foods	Hardly	Count (ratio in %)	233 (15.17)	408 (26.56)	187 (12.17)	6.61 (2)	0.037 *	B > C
		Modified residual	−0.23	2.21	−2.3			
	Almost	Count (ratio in %)	203 (13.22)	309 (20.12)	196 (12.76)			C > B
		Modified residual	0.23	−2.21	2.3			
My child avoids processed foods	Hardly	Count (ratio in %)	274 (17.84)	447 (29.10)	196 (12.76)	15.44 (2)	<0.0001 ***	B > C
		Modified residual	1.58	1.98	−3.93			
	Almost	Count (ratio in %)	162 (10.55)	270 (17.58)	187 (12.17)			C > B
		Modified residual	−1.58	−1.98	3.93			
My child avoids fried or creamy foods and other fat-rich foods	Hardly	Count (ratio in %)	301 (19.60)	493 (32.10)	217 (14.13)	19.05 (2)	<0.0001 ***	B > C
		Modified residual	1.67	2.27	−4.36			
	Almost	Count (ratio in %)	135 (8.79)	224 (14.58)	166 (10.81)			C > B
		Modified residual	−1.67	−2.27	4.36			

Variables of Significance (* *p* ≤ 0.05, ** *p* ≤ 0.01, *** *p* ≤ 0.001); N.S. = not significant (*p* > 0.05).

## Data Availability

Not applicable.

## Appendix A

Questions on symptoms of fatigue in children

1. My child seldom engages in physical play
2. My child often yawns during the day
3. My child often looks sleepy
4. My child becomes tired easily
5. My child often becomes irritated
6. My child often has difficulty remaining calm
7. My child often has difficulty concentrating when playing
8. My child often has difficulty sitting still
9. My child often experiences headaches and stomach pain
10. My child often looks unwell

## Appendix B

Questions on children’s sleep quality

1. Ability to fall asleep within 15 min
2. Sleep through the night
3. No sleep talking
4. No enuresis during sleep
5. No sleep terrors
6. No sleep walking
7. Nocturnal sleep for 10–11 h on weekdays
8. Nocturnal sleep for 10–11 h on weekends and holidays
9. Going to bed before 9 p.m. on weekdays
10. Going to bed before 9 p.m. on weekends
11. Waking up before 7 a.m. on weekdays
12. Waking up before 7 a.m. on weekends
13. Regular sleep schedule

## Appendix C

Questions on children’s dietary habits

1. My child eats breakfast every day
2. My child eats three meals of a consistent quantity punctually every day
3. My child chews slowly
4. My child avoids eating late-night snacks
5. My child is not a picky eater
6. My child eats foods from the six major groups every day
7. My child eats at least three servings of vegetables every day
8. My child eats at least two fruits every day
9. My child drinks at least 1500 mL of boiled water every day
10. My child avoids eating snacks
11. My child avoids sodas and sugar-sweetened beverages
12. My child avoids Western-style fast foods such as hamburgers and French fries
13. My child avoids instant noodles and other instant foods
14. My child avoids processed foods
15. My child avoids fried or creamy foods and other fat-rich foods
